# Cigarette Affordability in China, 2006–2015: Findings from International Tobacco Control China Surveys

**DOI:** 10.3390/ijerph16071205

**Published:** 2019-04-03

**Authors:** Nigar Nargis, Rong Zheng, Steve S. Xu, Geoffrey T. Fong, Guoze Feng, Yuan Jiang, Yang Wang, Xiao Hu

**Affiliations:** 1 American Cancer Society, 555 11th Street NW, Suite 300, Washington, DC 20004, USA; 2 School of International Trade and Economics, University of International Business and Economics, Beijing 100029, China; rosezheng@uibe.edu.cn (R.Z.); wangyang1989shida@163.com (Y.W.); xiaoxiaouiber@126.com (X.H.); 3 Department of Psychology, University of Waterloo, Waterloo, ON N2L 3G1, Canada; s4xu@uwaterloo.ca (S.S.X.); geoffrey.fong@uwaterloo.ca (G.T.F.); 4 Ontario Institute for Cancer Research, Toronto, ON M5G 0A3, Canada; 5 Chinese Center for Disease Control and Prevention (China CDC), Beijing 102206, China; fengguoze@hotmail.com (G.F.); jiangyuan88@vip.sina.com (Y.J.)

**Keywords:** cigarette consumption and affordability, socio-economic pattern, public health

## Abstract

China is world’s largest market of machine-made cigarettes. In 2015, more than 315 million or around 26.9% of the adult population in China were smokers—50.6% among men and 2.2% among women. Growing affordability of cigarettes led to increased cigarette consumption in China to the detriment of public health. This study investigated whether the level and growth in cigarette affordability in China was equally shared by smokers from all demographic and socio-economic statuses (SES) and across all price tiers of cigarette brands. The data came from the urban smoker sample (≥18 years) of the International Tobacco Control China Surveys conducted in five waves over 2006–2015. Cigarette affordability was measured by Relative Income Price—percentage of per capita household income needed to purchase 100 cigarette packs of the last purchased brand. Overall and group-specific trends in affordability by age, gender, SES (e.g., income, education, and employment status), and price tiers were analyzed using generalized estimating equations method. Cigarette affordability was higher among older, female, and higher-SES smokers, and for cheaper brands. It increased overall and across all groups over time. The increase was significantly larger among younger and lower-SES smokers, a trend that poses an added challenge to tobacco control and health equity. To reduce cigarette affordability and consumption among these vulnerable groups, a uniform specific excise system should be introduced in place of the existing tiered ad valorem excise. The specific excise should be periodically adjusted to inflation and per capita income growth observed among younger and lower-SES people, who can potentially experience faster income growth than the national average. The excise tax policy can also be complimented with minimum price regulations and restrictions on price promotions.

## 1. Introduction

China is the world’s largest market of machine-made cigarettes, consuming about 2.5 trillion cigarettes per year which accounted for 44% of global cigarette sale in 2017 [1]. This is more than half of what low- and middle-income countries (LMICs) including China consume together. Between 2003 and 2014, global (excluding China) cigarette consumption decreased by 27% from 1082 to 792 pieces per adult aged 15 and older, while increasing by 19% from 1980 to 2346 pieces per adult in China [1,2]. In 2000, 30.2% of the adult population (age 15 and above) in China were smokers—56.5% among men and 2.9% among women [3]. Although gradually decreasing over time, adult smoking prevalence in China remained alarmingly high at 26.9% as of 2015—50.6% among men and 2.2% among women [3]. The current smoking prevalence among men in China is among the highest in the world. 

The health, economic, and social costs of the high level and growing trend of cigarette consumption in China are enormous [4]. In 2000, it was estimated that the morbidity and premature mortality caused by smoking-attributable diseases led to USD 3.0 billion in health care costs and USD 5.8 billion in productivity loss (2012 prices) [5]. By 2012, the estimates soared to USD 13.6 billion in health care costs and USD 48.9 billion in productivity loss (2012 prices) [6]. The total cost of smoking, including health care costs and productivity loss, escalated seven-fold as a direct consequence of increasing number of smokers in the population.

The upward trend in cigarette consumption in China turned around only recently in 2015 when China’s Ministry of Finance announced the introduction of a specific excise tax at the wholesale level at 0.10 yuan (0.02 USD) per pack of 20 cigarette sticks, alongside an increase in the wholesale ad valorem tax rate from 5% to 11%. It led to an average increase in the retail price of cigarettes by 11% and contributed to reduction in cigarette consumption in the years 2015 and 2016 [7]. Compared to 2014, per adult cigarette consumption registered 9% decrease in China by 2017, while global consumption (excluding China) decreased further by 11% over the same period [1].

The sustained rise in cigarette consumption in China until 2014 was largely attributable to a continuous increase in cigarette affordability observed for longer than the past two and a half decades [8,9,10,11]. The rise in affordability was driven by the unprecedented income growth (at nearly 10% per annum) outpacing the growth in cigarette prices [11]. It was accentuated by tobacco industry interference through aggressive marketing strategies that continued until the development of smoke-free legislation and a ban on all forms of tobacco advertising, promotion, and sponsorship in 2014 [12]. The state-owned manufacturer, China National Tobacco Company (CNTC), and the State Tobacco Monopoly Administration (STMA) in charge of tobacco regulation are both government institutions and pose a major conflict of the public health interest of tobacco control with the business motive of tobacco production and sale.

Existing indices of affordability relating the price of cigarettes to a national level measure of income (e.g., per capita GDP, per capita disposable income) have enabled global comparison and determination of country-level trends of cigarette affordability [13]. Measures of affordability are also crucial for periodic evaluation and adjustment of tax levels, in line with the guidelines for implementation of Article 6 on price and tax measures to reduce the demand for tobacco under the World Health Organization Framework Convention on Tobacco Control (WHO FCTC) [14]. However, in the aggregate level measure, it is unclear how affordability of cigarettes varies across different socio-economic groups within a population and how the growth in affordability is shared by each sub-group. In view of high-level and continued income inequality in China [15], it is important to assess affordability based on individual or household level income. Tracking affordability by socio-economic status (SES) can not only indicate which sub-groups are experiencing increased ability to purchase tobacco products contributing to increased tobacco consumption at the national level and hence are at greater risk of tobacco-induced diseases, but also highlights the focus for future tax policy changes to deliver more equitable health outcomes.

Besides income disparity, significant price disparity may exist across cigarette brands which can affect the affordability of cigarettes depending on the choice of brands. Cigarette price disparity in China is attributed to the availability of more than 100 brands of cigarettes. In 2015, there were about 870 variants by price, packaging and quality of 89 brand families available in the market, with retail prices varying from 2.5 yuan (0.40 USD) to 100 yuan (16.06 USD) per pack [7]. Such a wide range of cigarette prices offers smokers the room for switching brands when affordability changes for the brands they usually smoke due to changes in price relative to their personal income. In China, it has been observed that lower income smokers are more likely to choose an economy brand because of its low price [16]. Thus, the matching of prices with income determines affordability at the individual level. For example, the ability of a low-income person to buy a cheap brand may be comparable to the ability of a medium-income person to buy a relatively expensive brand. 

Due to wide variation in cigarette price and income in China, affordability based on aggregate level price and income measures can bias the estimate of the mean percentage of income needed to purchase a given quantity of cigarettes. Besides, China experienced a significant increase in the market shares of premium and mid-priced brands and a decrease in the market share of economy brand cigarettes since the CNTC began its premiumization strategy to promote more expensive brands as better quality and less harmful cigarettes in 2009 [17]. The shifting composition of cigarette brands in different price categories may not be reflected in a summary measure of cigarette prices which can bias the trend in affordability.

Using individual level data on the purchase price of cigarettes and income, this study examined the distribution of cigarette affordability among a cohort of adult Chinese urban smokers. It identified the demographic and socio-economic patterns of affordability and its trend over time. In addition, the level and trend of affordability of cigarettes was examined by price categories of cigarette brands to enhance the understanding of the shift in brand composition of the cigarette market in China. 

The increase in overall cigarette affordability observed in this paper aligns with the findings from previous research. This paper additionally found that the growth in affordability was significantly larger among younger and lower-SES smokers, posing an added challenge to tobacco control in China. To target reduction in affordability and cigarette consumption among these vulnerable groups, the inflation-adjusted tax and price increases should be indexed to per capita income growth experienced by younger and lower-SES people, beyond national level per capita income growth.

## 2. Materials and Methods

The data for the analysis in this paper came from the urban smoker sample of the International Tobacco Control Policy Evaluation Project—China Surveys (the ITC China Survey). The ITC China Survey is a face-to-face longitudinal prospective cohort survey of a representative sample of adult (≥18 years) smokers and non-smokers. The fives waves of the ITC China Survey spanned from 2006 to 2015 (Waves 1: 2006, 2: 2007–2008, 3: 2009, 4: 2011–2012, and 5: 2013–2015). The Wave 1 to 4 surveys were conducted in six cities in China—Beijing, Changsha, Guangzhou, Shanghai, Shenyang, and Yinchuan. Kunming was added as the seventh city at Wave 3. The Wave 5 Survey was conducted in five cities that were included in previous waves (Beijing, Guangzhou, Kunming, Shanghai, and Shenyang), and in five new rural areas (Changzhi, Huzhou, Tongren, Yichun, and Xining). From each survey location, about 800 smokers were recruited using a stratified multistage cluster sampling design.

Each city was treated as a stratum. Within each city, the first stage clusters were the districts and the second stage clusters were the residential blocks. To ensure representativeness of individual observations on smokers in each wave, cross-sectional survey weights were used in the pooled analysis that accounted for the complex survey design. The retention rate of sample from one wave to the next was over 80%. The loss of sample due to attrition was replenished with respondents with comparable characteristics. The cross-sectional sampling weights accounted for the modifications of sample design due to selection of replenishment sample and changes in sampling clusters across survey waves. The details of the sampling and survey methods can be found elsewhere [18,19]. 

Ethics approval was obtained from the Office of Research Ethics (ORE) at the University of Waterloo (Waterloo, Canada), and the Institutional Review Boards (IRB) at Roswell Park Comprehensive Cancer Center (Buffalo, USA), Cancer Council Victoria (Melbourne, Australia), and the Chinese Center for Disease Control and Prevention (Beijing, China).

Approval Committee: University of Waterloo Human Research Ethics Committee, China CDC IRB, Cancer Council Victoria (Australia) IRB. 

Date and identification code:

- University of Waterloo (Canada) ORE # 12539—March 22, 2006
- University of Waterloo (Canada) ORE # 15305—January 1, 2009
- University of Waterloo (Canada) ORE # 17014—February 8, 2011
- Cancer Council Victoria (Australia) IRB: IER0803—October 14, 2010
- Chinese Center for Disease Control and Prevention IRB 201114—August 8, 2011
- Chinese Center for Disease Control and Prevention IRB 201325—June 25, 2013.

Cigarette affordability in this paper was measured by Relative Income Price (RIP), defined as the percentage of per capita income required to purchase 100 packs of 20-piece cigarettes. RIP was introduced as a national level measure and modified as an individualized measure in subsequent research [20,21]. The individualized affordability measure was constructed using self-reported price and per capita household income variables.

The analysis in this paper was limited to factory-made cigarettes that were smoked by 96.4% of current smokers in China in 2015 [22]. Other tobacco products that were in use include roll-your-own cigarettes, pipes, cigars, water pipes, and e-cigarettes. These tobacco products were not covered in the present analysis as the ITC data on purchases and prices were available for factory-made cigarettes only.

In all waves of ITC China Survey, smokers reported the total price paid, the total number of packs of 20 sticks and the brand of cigarettes bought in their last purchase. The self-reported unit price per pack of 20 cigarettes was determined by dividing the total price paid by the number of packs purchased for a specific brand. The self-reported prices were compared with the CNTC’s recommended retail prices by brands. Observations with self-reported prices exceeding the maximum recommended retail prices in the corresponding years were dropped.

The quantity of cigarettes bought in the last purchase may not reflect a smoker’s daily cigarette consumption or smoking intensity. For example, smokers may be encouraged to make bulk purchases when they are offered price discounts or promotions on purchases in larger quantities. An appropriate indicator of smoking intensity is the number of cigarettes smoked per day which was reported by the smokers in the ITC China survey. The number of cigarettes smoked per day was multiplied with the unit price to obtain smokers’ daily spending on cigarettes. The daily spending was converted to annual cigarette expenditure (CIGEXP) by multiplying by 365 and then expressed as a share of annual household income (CIGEXPSHARE). While RIP is an indicator of the ability to purchase a given amount (100 packs) of cigarettes, CIGEXPSHARE represents the actual spending on cigarette purchase made by smokers as a share of their household income.

Smokers reported their monthly household income belonging to six income groups: <1000 yuan (161 USD), 1000–2999 yuan (161-482 USD), 3000–4999 yuan (482–803 USD), 5000–6999 yuan (803–1124 USD), 7000–8999 yuan (1124–1445 USD), and 9000 yuan (1445 USD) and above. The mean annual household income was imputed corresponding to each income range based on data from China Household Finance Survey (CHFS) 2011 (Appendix A Table A1). The mean household income was then divided by household size to obtain per capita household income for each respondent. 

In the first three waves, respondents reported the number of adult male and female members of their households. They also reported whether any child was living in the household. Based on the child dependency ratio of 22.3% (2010 Chinese Census) and the number of adults in household, the number of children was estimated for the households of the respondents who reported to have any child living in the household. The number of adults and children were then added to estimate the size of the household. 

In Waves 4 and 5, respondents were not asked about the number of adult household members. The per capita household income in these two waves was assigned to individuals from the mean of per capita income values observed in the first three waves for respective household income group in the lower five income groups (Appendix A Table A1). The mean per capita annual household income values for the lower five income groups for the years 2012–2015 were not adjusted for inflation or income growth. The growth in household income for the lower five income groups was reflected only in the upward movement of respondents to higher income groups and vice versa. For the open-ended top income group with a monthly household income 9000 yuan (1445 USD) and above, the mean per capita household income (64,861 yuan or 10,038 USD) imported from the CHFS 2011 was adjusted for inflation and per capita income growth to obtain the nominal income values for respective years (Appendix A Table A1). 

Using the per capita household income constructed in the abovementioned method, RIP for each individual smoker, i, in survey wave, t, was measured as the ratio:

RIP _it_ = 100 x Price per pack of 20 cigarette sticks _it_ / Per capita annual household income _it_

The higher the proportion of income required to purchase 100 packs of cigarettes, the lower the affordability of cigarettes. As such, higher RIP would indicate lower affordability and vice versa.

In addition to this outcome variable of interest, we included several demographic and socio-economic characteristics of smokers, such as age, gender, level of education, employment status, the type of cigarette brands purchased by respondents last, city of residence, and the waves/years of interview. 

We analyzed the trend in overall cigarette affordability by using the following model in Equation (1):
RIP _it_ = α_0_ + α_1_ AGE(36–55) _it_ + α_2_ AGE(55+) _it_ + α_3_ FEMALE _i_ + α_4_ HIGH-INCOME _it_ + α_5_ HIGH-EDU _it_ + α_6_ EMPLOYED _it_ + Σ_j_ α_7j_ TIER _itj_ + Σ_k_ α_8k_ CITY _itk_ + Σ_l_ α_9l_ WAVE _il_ + u _it_(1)
where AGE(36–55) = 1 if individual age was in the range 36 to 55 and 0 otherwise, AGE(55+) = 1 if individual age was over 55 and 0 otherwise, FEMALE = 1 if a smoker was female and 0 otherwise, HIGH-INCOME = 1 if per capita monthly household income was above the ninth decile for urban households, HIGH-EDU = 1 if individuals had above high school level education and 0 otherwise, EMPLOYED = 1 if individuals has job and 0 otherwise referring to those without a job, student, or retired, TIER_j_ = 1 if a smoker reported the cigarette brand of last purchase in tier j and 0 otherwise, CITY_k_ =1 if a smoker reported the city of residence in city k and 0 otherwise, WAVE_l_ = if the observation belonged to survey-wave l and 0 otherwise, and u_it_ is unobserved random error term. The coefficients of the WAVE variables indicate the trend in overall cigarette affordability.

We analyzed the patterns and trends in cigarette expenditure (CIGEXP) and cigarette expenditure share in household income (CIGEXPSHARE) by using the following models in Equations (2) and (3):
CIGEXP _it_ = β_0_ + β_1_ AGE(36–55) _it_ + β_2_ AGE(55+) _it_ + β_3_ FEMALE _i_ + β_4_ HIGH-INCOME _it_ + β_5_ HIGH- EDU_it_ + β_6_ EMPLOYED _it_ + Σ_j_ β_7j_ TIER _itj_ + Σ_k_ β _8k_ CITY _itk_ + Σ_l_ β _9l_ WAVE _il_ + e _it_(2)
CIGEXPSHARE it = θ_0_ + θ_1_ AGE(36–55) _it_ + θ_2_ AGE(55+) _it_ + θ_3_ FEMALE _i_ + θ_4_ HIGH-INCOME _it_ + θ_5_
HIGH-EDU _it_ + θ_6_ EMPLOYED _it_ + Σ_j_ θ_7j_ TIER _itj_ + Σ_k_ θ_8k_ CITY _itk_ + Σ_l_ θ_9l_ WAVE _il_ + v_it_(3)

The National Bureau of Statistics of China classifies households into low, lower-middle, middle, upper-middle, and high-income status by income quintiles. Following this classification, smokers were classified into high-income status if their per capita monthly household income was above the ninth income decile for urban households in corresponding years available from the National Bureau of Statistics of China (Appendix A Table A2). Smokers with a per capita household income below the ninth decile were classified into low-income status. 

Cigarette brands in China are categorized into five price tiers based on producer price, with the most expensive or premium brands in Tier I and the cheapest brands in Tier V (Table 1). Details of the determination and classification of brands reported in ITC China Surveys are available elsewhere [17]. As shown in Table 1, tax rates vary depending on price tiers. Preferential tax rates offered to cheaper brands in Tiers III, IV, and V create a significant price differential across brand categories and affects the affordability of different brands. We therefore controlled for the price tiers in this model to allow for the effect of a tiered tax structure.

In the next step, we estimated the trend in cigarette affordability and expenditure by each classification of smokers’ characteristics using interaction terms of survey year with respective group variable. We ran separate regression for the interaction of time trend with each classification controlling for the remaining characteristics. The year-to-year comparison of RIP, CIGEXP, and CIGEXPSHARE was made with reference to 2007–2008 to identify the difference in affordability and spending on cigarettes immediately before and after the tax increase in 2009. As it will be evident later in the Results section, a significant increase in cigarette price and decrease in cigarette affordability occurred across all brands between 2006 and 2007–2008, when tax rates remained unchanged. The selection of the year 2007–2008 as a reference point was meant to isolate the trend in affordability and cigarette expenditures from the effect of price increases that were not induced by the tax increase in 2009.

The trend in affordability and expenditure by smokers’ sub-groups was measured by the coefficients of the interaction term of wave variable with corresponding characteristics. The contrast of the trend in affordability and cigarette expenditure across sub-groups was tested for statistical significance up to a 10% level. 

Since ITC survey provides panel data, ordinary least squares (OLS) estimation of Equations (1), (2) and (3) run over pooled observations of data from fives waves of the survey can generate biased estimates due to the existence of multiple observations on the same individual and within person correlated errors across waves. Hence, Equations (1), (2) and (3) were estimated using generalized estimating equations (GEE) method (xtgee in STATA V.13) that fits population-averaged panel-data model. The robustness of results was checked using both OLS and GEE methods. 

## 3. Results

### 3.1. Study Sample

The initial study sample consisted of all urban adult smoker respondents from the five waves. The sizes of survey sample and selected sample in each wave of survey are provided in Table 2. The total number of observations on urban smokers pooled from Waves 1–5 was 24,664. All nonsmoker respondents in Waves 1–4 and the rural smoker and nonsmoker respondents in Wave 5 were excluded in the first stage of the study sample selection. In the second stage, former urban smoker respondents (quitters) in Waves 2–5 were excluded and only current smokers were retained giving a total of 23,170 observations. In the third stage, the cases with missing values in the self-reported price and household income variables and the covariates used in the multivariate analysis were dropped. The final analytical sample consisted of 16,422 observations on 8578 individual smokers (both daily and occasional). As Table 2 shows, majority of the analytical samples were from the age group 36–55, were male, belonged to low-income households, had higher than high school level education, were employed, and smoked mid-price cigarette brands in price tier III at the time of the survey. The classification of the analytical sample into low- and high-income categories in 90:10 ratio indicates that the high-income category belongs to the top income decile in the sample, which is consistent with the urban income classification at the national level based on data from the National Bureau of Statistics (NBS) China (Appendix A Table A2). 

### 3.2. Trend in Overall Cigarette Affordability

On average, RIP decreased from 11.1% to 5.8% between 2006 and 2015 suggesting long-term increasing trend in affordability over this period (Table 3). The results of GEE estimation of equation (1) reported in Table 4 indicate that overall cigarette affordability decreased between 2006 and 2007–2008 (RIP 1.56 percentage points (pp) less in 2006 than in 2007–2008), then increasing gradually in subsequent waves (RIP 0.92 pp less in 2009, 3.91 pp less in 2011–2012, and 5.36 pp less in 2013–2015 compared to 2007–2008). 

The year-on-year decrease in cigarette affordability between 2006 and 2007–2008 is attributable to a significant hike in inflation-adjusted cigarette price across all price tiers in that year, which tapered off in subsequent years, except for the brands in the lowest price tier—V (Table 3). It follows that affordability increased significantly from 2007–2008 to 2013–2015, more so in the upper four price tiers than in the bottom tier (Table 3).

### 3.3. Cross-Sectional Variation in Cigarette Affordability by Smokers’ Characteristics

The GEE estimates in Table 4 demonstrate that there is measurable difference of cigarette affordability across different demographic and socio-economic groups of smokers, price categories of cigarette brands, and location of residence. It is significantly higher among older smokers (RIP 3.00 pp less for age group 55+) compared to the young adults (age group 18–35). Female smokers can afford cigarettes more than males (RIP 0.88 pp less). Affordability is higher among high-income (RIP 7.08 pp less than low-income), high-educated (RIP 1.90 pp less than low-educated) and employed (RIP 2.94 pp less than not employed) smokers. Cheaper cigarettes are more affordable than the expensive brands (RIP 9.32 pp less for the cheapest tier V, 7.70 pp less for the second cheapest tier IV, 5.35 pp less for the third cheapest tier III, and 2.58 pp less for the fourth cheapest tier II compared to the most expensive brands in tier I). Cigarettes are generally less affordable in the six cities, e.g., Shenyang (RIP 3.30 pp higher), Shanghai (RIP 1.94 pp higher), Changsha (RIP 4.39 pp higher), Guangzhou (RIP 1.60 pp higher), Kunming (RIP 3.97 pp higher), and Yinchuan (RIP 3.48 pp higher), compared to Beijing.

### 3.4. Trends in Cigarette Affordability by Smokers’ Characteristics

The RIP predicted from GEE estimation interacted by age, gender, household income status, education, employment status, and price tier of brands are presented in Table 5. The results indicate that cigarette affordability increased significantly across all three age groups of smokers, e.g., 18–35, 36–55, and 55+ years. The increase in affordability was largest among the middle age group of smokers (36–55) by 6.1 pp. The increase was significantly different between the youngest (18–35) and the oldest adults (55+) by 1.6 pp (*p*-value = 0.0820). Male and female smokers shared the same rate of change in cigarette affordability by 5.4 pp. 

While smokers from both low- and high-income households enjoyed an increase in cigarette affordability, the increase for low-income smokers was twice as large as for high-income smokers. Table 5 shows that in 2007–2008 a low-income smoker needed on average 12.3% of his/her per capita household income to purchase 100 packs of cigarettes of the last purchased brand. In 2013–2015, 100 packs of cigarettes of the last purchased brand cost 6.5% of per capita household income of low-income smokers on average, which is 5.8 pp less than the 2007–2008 level of RIP. For high-income smokers, 100 packs of cigarettes cost 4.1% of per capita income in 2007–2008; the cost fell to 1.2% in 2013–2015, suggesting a smaller increase in affordability by 2.9 pp among high-income smokers. The difference-in-difference in RIP between low- and high-income smokers from 2007–2008 to 2013–2015 given by 2.9 pp (−5.8–(−2.9)) is statistically significant (*p*-value = 0.0050).

Affordability increased for smokers with both low (less than high school) and high (high school and above) levels of education, the increase being higher for the low-education group. The difference-in-difference in affordability (1.9 pp) is statistically significant (*p*-value = 0.0000) (Table 5).

Both employed and not employed (e.g., students, retired, unemployed, homemakers) smokers experienced a significant increase in cigarette affordability. The increase was statistically significantly higher for the not-employed smokers (6.3 pp) compared to the employed smokers (4.7 pp) (Table 5).

Affordability increased significantly for cigarette brands in all price tiers. The increase in affordability was statistically significantly larger for the premium brands in the top tier I compared to the bottom four tiers (Table 5).

### 3.5. Trends in Cigarette Expenditure and Its Share in Smokers’ Houesehold Income

Annual cigarette expenditure per smoker varied from 3176 yuan (510 USD) accounting for 14.1% of smokers’ household income in 2006 to 4,410 yuan (708 USD) accounting for 7.4% of smokers’ household income on average. The results of GEE estimation of equation (2) on cigarette expenditure per smoker reported in Table 6 show that cigarette expenditure was considerably higher (by 898.48 yuan in 2015 constant prices) in 2007–2008 compared to 2006, which can be associated with a significant hike in inflation-adjusted cigarette prices across all price tiers in that year (Table 3). Cigarette expenditure stabilized in subsequent years, as reflected in much smaller increases (107.65 yuan in 2009, 133.24 in 2011–2012, and 20.79 yuan in 2013–2015) (Table 6), which is attributable to sluggish price increases over this period (Table 3).

The share of cigarette expenditure in household income, on the other hand, plummeted after 2007–2008 by 0.01 pp in 2009, 0.03 pp in 2011–2012, and 0.05 in 2013–2015 compared to 2007–2008 (Table 7). This finding is consistent with stable cigarette expenditure accompanied by high income growth, which caused growing affordability of cigarettes as shown in Table 3 and Table 4.

### 3.6. Trends in Cigarette Expenditure and Its Share in Smokers’ Houesehold Income by Smokers’ Characteristics

The GEE estimates of annual cigarette expenditure by smokers’ characteristics in Table 8 show that the level of cigarette expenditure was generally higher among higher income group. However, no significant trend in cigarette expenditure was observed by age group, gender or SES. The changes in annual cigarette expenditure between 2007–2008 and 2013–2015 were not statistically significant.

The share of cigarette expenditure in smokers’ household income, however, decreased significantly across all demographic and socio-economic groups (Table 9), in line with the increase in affordability observed earlier. The decrease was significantly larger among low educated and non-employed smokers. The significantly faster reduction in the share of cigarette expenditure in household income coupled with unchanged cigarette expenditure for the low educated and non-employed smokers shown in Table 8 indicates that these low-SES smokers experienced faster income growth than their high-SES counterparts. 

## 4. Discussion

Global evidence shows that per capita cigarette consumption is negatively associated with cigarette affordability. Growing affordability of cigarettes has been contributing to increasing cigarette consumption in many LMICs, posing serious challenges in tobacco control and an enormous threat to global health [20,23]. China is no exception to this phenomenon. The overall growth in cigarette affordability in China is attributable to faster growth in per capita income than the price of cigarettes.

Using five waves of longitudinal data of cigarette purchases of individual smokers in China spanning from 2006 to 2015, this paper observes that overall cigarette affordability increased significantly since 2007–2008 despite an increase in the excise tax on cigarettes in 2009. The STMA controls the price of cigarettes through the supply chain from production to wholesale to retail levels. Following the tax increase in 2009, STMA significantly reduced the profit margin at the wholesale level and did not allow the wholesale and retail prices to increase [24]. As a result, between 2009 and 2011–2012, cigarette prices decreased by 1–3% for the brands in tiers I to IV and increased by 5% for only tier V (Table 2). Over this period, national per capita disposable income was increasing annually at a rate above 10% [25], contributing significantly to people’s purchasing power, consequent upon an increase in cigarette affordability and consumption in the following years. 

The Chinese government raised cigarette excise tax again in 2015. This time, STMA allowed cigarette prices to increase. An early assessment reveals that this tax increase led to 7% increase in average retail price of cigarettes and 16% increase in the retail price of the cheapest category of cigarette brands in 2015 after adjustment for inflation, and 7.8% reduction in annual cigarette sales between 2014 and 2016 [7]. The reduction in cigarette consumption is partly attributable to the fact that the price increase following the tax increase more than offset the effect of per capita disposable income growth that slowed down to less than 7% per year [25]. The contrasting evidence of the impact on cigarette consumption following the tax increases in 2009 and 2015 suggests that tax increases need to be translated into price increases large enough to outpace the growth of people’s income and purchasing power and reduce cigarette consumption.

Smokers in higher SES (by per capita household income, individual education, or employment status) demonstrated greater affordability of cigarettes than those with lower SES. This finding is consistent with the observation that tobacco consumption is higher among richer than the poorer people in China [25,26,27].

The trend in RIP by sub-groups of smokers shows that smokers from low-income households, low education status and smokers without employment experienced greater increase in affordability. It indicates convergence in cigarette affordability across different SES in China over time. The faster growth in cigarette affordability among the low-SES population observed in this study is consistent with economic theories and the empirical finding in China based on the ITC China Survey that price sensitive lower-SES people are more prone to price minimizing behaviors, such as switching to cheaper brands, bulk purchases, use of price promotions and discounts, purchasing from tax-free or lower tax locations, and the like. [16,28] Besides, low educated and non-employed smokers maintained a stable level of cigarette expenditure while the share of cigarette expenditure in their household income decreased at a faster rate than high-SES smokers. These results suggest that the low-SES smokers also experienced disproportionately larger income growth, leading to faster growth in their cigarette affordability.

Faster growth in cigarette affordability is likely to encourage cigarette consumption among the low-SES population further. Smoking-related expenses, such as direct household spending on cigarettes and excessive medical spending caused by smoking-induced diseases, have been responsible for impoverishing a significant proportion of low-income households into poverty in China [29]. Rampant growth in cigarette affordability and consumption among low-SES population will exacerbate the incidence of poverty due to smoking contributing to greater health and economic inequality in the longer run.

The convergence in cigarette affordability is also observed between younger and older adults. Faster growth of affordability of cigarettes among younger adults is likely to encourage smoking initiation and undermine the prospect of making significant health gains through cessation at a young age [30]. Faster growth of cigarette affordability and consumption among low-SES and younger populations may pose added challenges to tobacco control in China. The heartening fact is that low-income and younger people are also more sensitive to tax and price increases [31]. Tobacco tax policy can, therefore, be harnessed to arrest the faster pace of rising affordability among these vulnerable groups of the population. 

Introducing a uniform specific excise would be more desirable than the existing tiered ad valorem excise tax structure to reduce affordability and consumption of cigarettes among low-SES and young people. The rationale is that an increase in specific tax would raise the price of cheaper brands, that are more likely to be consumed by low-SES and younger smokers, relative to higher-price brands. The specific excise, however, needs to be adjusted periodically to keep pace with inflation and growing income [14]. The findings from this study imply that it is not sufficient to index inflation-adjusted tax and price increases to per capita income growth at the national level. The indexation needs to consider per capita income growth experienced by low-SES and younger people, the population sub-groups who are likely situated in the trajectory of upward income mobility and experience faster income growth compared to the national average. 

In addition, minimum price regulations and restrictions on price promotions targeting younger and low-SES population can be applied to compliment excise tax policy in raising average cigarette prices and reducing price variability across different brands [32]. These measures are likely to arrest the growing trend in cigarette affordability among low-SES and younger smokers and prevent disparities in tobacco consumption and health from widening.

President Xi Jinping’s Healthy China Plan 2030, announced in October 2016, targets reduction of adult smoking prevalence from the current 27.7% to 20% by the year 2030 [33]. The second draft of the Essential Health and Health Promotion Law recognizes tobacco taxation and pricing as an important tobacco control measure to achieve this target [34]. The findings from the current research would inform the policymakers in China and help mold tobacco taxation and pricing measures to serve equity in health.

One major limitation of this study is that the results of the analysis are representative of only the urban adult smokers in China. It is important to recognize the lack of representation of the rural smokers in this paper in view of China’s high degree of income inequality particularly with respect to the rural–urban gap. The ratio of per capita disposable income of urban to rural population has been greater than 3 during 2001–2016 [25]. Exploring the trend in affordability by SES among the rural population can be subject to future research.

Second, the survey period under Wave 5 (November 01, 2013 to July 24, 2015) overlapped to some extent with the implementation of the increase in excise tax on cigarettes since May 2015. Nevertheless, the assessment of the effect of this tax increase on cigarette affordability and expenditures were not feasible due to lack of enough observations after the policy change. 

## 5. Conclusions

Growing affordability of cigarettes has led to continuous growth in cigarette consumption in China. Faster growth of cigarette affordability among younger and low-SES population poses added challenge to tobacco control in China. Tobacco tax policy can be harnessed to curb the faster pace of rising affordability among these specific groups of the population. A uniform, specific excise system should be introduced in place of the existing tiered, ad valorem excise for cigarettes. The specific excise should be periodically adjusted to inflation and per capita income growth. However, it is not sufficient to index inflation-adjusted tax and price increases to per capita income growth at the national level to reduce group-specific affordability and consumption of cigarettes. The indexation needs to consider per capita income growth experienced by younger and lower-SES people, the population sub-groups who are likely situated in the trajectory of faster income growth compared to the national average. The excise tax policy can also be complimented with minimum price regulations and restrictions on price promotions targeting young and low-SES populations.

## Figures and Tables

**Table 1:** Price tiers and excise tax structure of cigarettes in China, 2001–2015.

Price/Tax →	Price Range, VAT Exclusive (yuan/per pack of 20 sticks)	Excise Tax		
Level ↓	2015	June 2001 to April 2009	May 2009 to April 2015	Effective from May 2015
**Producer**				
Specific excise				
yuan/pack of 20 sticks	All	0.06	0.06	0.06
USD/pack of 20 sticks	All	0.01	0.01	0.01
Ad valorem excise (% of producer price)				
Tier I	>10	45%	56%	56%
Tier II	7–10	45%	56%	56%
Tier III	3–7	45%	36%	36%
Tier IV	1.65–3	30%	36%	36%
Tier V	<1.65	30%	36%	36%
**Wholesale**				
Specific excise (yuan/pack of 20 sticks)	All	-	-	0.10
Ad valorem excise (% of wholesale price)	All	-	5%	11%

Note: The specific excise is converted to USD equivalent using the exchange rates 1 USD = 6.83 yuan in 2009 and 1 USD = 6.23 yuan in 2015.

**Table 2:** Summary of sample selection and characteristics of the analytical sample.

Sample Information	Wave 1	Wave 2	Wave 3	Wave 4	Wave 5	Pooled
	2006	2007–2008	2009	2011–2012	2013–2015	
**Survey sample size**						
Smoker (Urban)	4815	4844	5585	5560	3860	24664
Non-smoker (Urban)	1270	1221	1417	1422	1049	6379
Smoker (Rural)					3957	3957
Non-smoker (Rural)					1014	1014
Included: Current smoker (Urban)	4815	4627	5211	5082	3435	23170
Size of final analytical sample	3214	3186	3849	3727	2446	16422
**Characteristics of analytical sample**						
**Age group (%)**						
18–35	10.5%	9.6%	14.2%	13.2%	12.4%	12.1%
36–55	60.0%	61.5%	57.0%	54.9%	50.7%	57.0%
55+	29.5%	29.0%	28.8%	32.0%	36.9%	30.9%
**Gender (%)**						
Male	95.5%	95.5%	95.7%	95.9%	95.0%	95.6%
Female	4.5%	4.5%	4.3%	4.1%	4.0%	4.4%
**Household income (%)**						
Low income	87.5%	95.9%	93.2%	92.7%	80.7%	90.6%
High income	12.5%	4.1%	6.8%	7.3%	19.3%	9.4%
**Education (%)**						
Low education	44.6%	43.1%	40.3%	39.6%	38.6%	41.3%
High education	55.4%	56.9%	59.7%	60.4%	61.4%	58.7%
**Employment status (%)**						
Not employed	36.6%	37.3%	36.8%	40.9%	44.3%	38.9%
Employed	63.4%	62.7%	63.2%	59.1%	55.7%	61.1%
**Smokers using brands by price tier (%)**						
I (most expensive)	11.3%	4.2%	7.6%	14.0%	19.9%	10.9%
II	31.5%	9.4%	2.8%	6.8%	9.6%	11.6%
III	48.1%	33.3%	47.9%	52.5%	55.7%	47.3%
IV	8.8%	39.9%	31.7%	20.7%	10.6%	23.2%
V (cheapest)	0.3%	13.3%	10.8%	5.9%	4.2%	7.0%
**City of residence (%)**						
Beijing	14.8%	15.9%	14.0%	12.7%	18.7%	14.9%
Shenyang	12.2%	17.3%	15.6%	15.3%	18.7%	15.6%
Shanghai	21.5%	21.3%	16.7%	16.4%	24.6%	19.7%
Changsha	22.2%	19.1%	14.3%	15.2%	-	14.9%
Guangzhou	14.5%	10.9%	7.7%	7.9%	13.7%	10.6%
Kunming	-	-	18.0%	16.8%	24.4%	11.7%
Yinchuan	14.8%	15.5%	13.7%	15.7%	-	12.7%

**Table 3:** Average self-reported price of cigarettes per pack of 20 pieces and average Relative Income Price (RIP) of cigarettes in China, 2006 to 2013–2015.

**Average Price per Pack of 20 Cigarettes (Yuan in 2015 constant prices)**						
**Tier**	**2006**	**2007–2008**	**2009**	**2011–2012**	**2013–2015**	
I	18.25	23.18	24.08	23.75	22.29	
II	9.05	12.39	13.92	13.47	14.35	
III	5.65	8.86	9.47	9.39	9.21	
IV	3.00	5.63	5.58	5.54	5.34	
V	2.53	3.20	3.47	3.66	4.98	
**Average Price per Pack of 20 Cigarettes (USD in 2015 constant prices)**						
**Tier**	**2006**	**2007–2008**	**2009**	**2011–2012**	**2013–2015**	
I	2.93	3.72	3.87	3.81	3.58	
II	1.45	1.99	2.24	2.16	2.30	
III	0.91	1.42	1.52	1.51	1.48	
IV	0.48	0.90	0.90	0.89	0.86	
V	0.41	0.51	0.56	0.59	0.80	
**Increase in Average Price (%)**						
**Tier**	**2006 to 2007–2008**	**2007–2008 to 2009**	**2009 to 2011–2012**	**2011–2012 to 2013–2015**		**Percentage change: 2007–2008 to 2013–2015**
I	27%	4%	−1%	−6%		−4%
II	37%	12%	−3%	7%		16%
III	57%	7%	−1%	−2%		4%
IV	87%	−1%	−1%	−4%		−5%
V	27%	8%	5%	36%		56%
**Average Relative Income Price (RIP, %)**						
**Tier**	**2006**	**2007–2008**	**2009**	**2011–2012**	**2013–2015**	**Percentage point change: 2007–2008 to 2013–2015**
I	13.2%	20.2%	17.9%	11.9%	7.7%	−12.5%
II	12.7%	13.5%	13.9%	9.9%	6.9%	−6.5%
III	11.4%	11.9%	11.1%	7.9%	5.5%	−6.4%
IV	7.5%	12.2%	10.0%	6.4%	5.0%	−7.2%
V	4.8%	7.2%	7.5%	4.1%	3.2%	−4.1%
Overall	11.1%	11.7%	10.5%	7.7%	5.8%	−5.9%

Note: The average prices per pack of 20 cigarettes were adjusted for inflation and expressed in 2015 prices using the Consumer Price Index for China.

**Table 4:** Generalized Estimating Equations (GEE) estimation of Relative Income Price (RIP) in China, 2006–2015.

	Coef.	Std. Err.	z	P>|z|	(95% Conf. Int.)	
**Age: Reference “Age 18–35”**						
36–55	0.14	0.28	0.52	0.61	−0.40	0.68
55+	−3.00	0.32	−9.35	0.00	−3.63	−2.37
**Gender: Reference “Male”**						
Female	−0.88	0.42	−2.10	0.04	−1.69	−0.06
**Household income: Reference “Low income”**						
High income	−7.08	0.28	−24.88	0.00	−7.64	−6.52
**Education: Reference “Low education”**						
High education	−1.90	0.18	−10.56	0.00	−2.25	−1.54
**Employment status: Reference “Not employed”**						
Employed	−2.94	0.20	−15.01	0.00	−3.32	−2.55
**Price tier of cigarettes: Reference “Tier I (most expensive)”**						
II	−2.58	0.35	−7.35	0.00	−3.27	−1.89
III	−5.35	0.28	−19.15	0.00	−5.90	−4.80
IV	−7.70	0.33	−23.40	0.00	−8.35	−7.06
V (cheapest)	−9.32	0.43	−21.79	0.00	−10.16	−8.48
**City of residence: Reference “Beijing”**						
Shenyang	3.30	0.31	10.57	0.00	2.69	3.91
Shanghai	1.94	0.31	6.22	0.00	1.33	2.55
Changsha	4.39	0.32	13.53	0.00	3.75	5.02
Guangzhou	1.60	0.35	4.63	0.00	0.92	2.28
Kunming	3.97	0.35	11.33	0.00	3.28	4.65
Yinchuan	3.48	0.33	10.47	0.00	2.83	4.14
**Survey wave: Reference “Wave 2 (2007–2008)”**						
2006	−1.56	0.27	−5.88	0.00	−2.08	−1.04
2009	−0.92	0.24	−3.87	0.00	−1.39	−0.45
2011–2012	−3.91	0.25	−15.91	0.00	−4.39	−3.43
2013–2015	−5.36	0.29	−18.67	0.00	−5.92	−4.80
**Constant**	18.66	0.51	36.35	0.00	17.65	19.66

Note: Coef. = Coefficient estimates; Std. Err. = Standard Error of Coefficient estimates; z = z statistic; P>|z| = *p*-value of z statistic; 95% Conf. Int. = 95% Confidence Interval of the Coefficient estimates.

**Table 5:** Trend in Relative Income Price (RIP) of cigarettes (predicted from GEE estimation) by age, gender, household income status, education, employment status and price tier of brands in last purchase of smokers in China, 2007–2008 to 2013–2015.

**RIP by Age Group**	**2006**	**2007–2008**	**2009**	**2011–2012**	**2013–2015**	**Change: 2007–2008 to 2013–2015**	***p*-value**	**Difference in Change over 2007–2008 to 2013–2015 in Comparison to Age Group 18–35**	***p*-value**
18–35	9.8%	12.6%	12.2%	8.5%	7.0%	−5.6%	0.0000		
36–55	11.5%	12.9%	11.4%	8.5%	6.8%	−6.1%	0.0000	−0.5%	0.5980
55+	7.4%	8.7%	8.7%	5.8%	4.7%	−4.0%	0.0000	1.6%	0.0820
**RIP by gender**	**2006**	**2007–2008**	**2009**	**2011–2012**	**2013–2015**	**Change: 2007–2008 to 2013–2015**	***p*-value**	**Difference in change over 2007–2008 to 2013–2015 between male and female**	***p*-value**
Male	10.1%	11.6%	10.7%	7.7%	6.2%	−5.4%	0.0000		
Female	9.2%	11.4%	9.4%	6.2%	6.0%	−5.4%	0.0000	−0.1%	0.9650
**RIP by household income status**	**2006**	**2007–2008**	**2009**	**2011–2012**	**2013–2015**	**Change: 2007–2008 to 2013–2015**	***p*-value**	**Difference in change over 2007–2008 to 2013–2015 between low- and high-income**	***p*-value**
Low-income	10.8%	12.3%	11.3%	8.3%	6.5%	−5.8%	0.0000		
High-income	2.8%	4.1%	3.5%	1.1%	1.2%	−2.9%	0.0040	2.9%	0.0050
**RIP by educational status**	**2006**	**2007–2008**	**2009**	**2011–2012**	**2013–2015**	**Change: 2007–2008 to 2013–2015**	***p*-value**	**Difference in change over 2007–2008 to 2013–2015 between low and high education**	***p*-value**
Low education	11.0%	13.1%	11.6%	8.8%	6.6%	−6.5%	0.0000		
High education	9.2%	10.3%	9.9%	6.7%	5.8%	−4.6%	0.0000	1.9%	0.0000
**RIP by employment status**	**2006**	**2007–2008**	**2009**	**2011–2012**	**2013–2015**	**Change: 2007–2008 to 2013–2015**	***p*-value**	**Difference in change over 2007–2008 to 2013–2015 between not employed and employed**	***p*-value**
Not employed	12.1%	13.6%	12.3%	9.3%	7.4%	−6.3%	0.0000		
Employed	8.6%	10.2%	9.5%	6.5%	5.5%	−4.7%	0.0000	1.6%	0.0040
**RIP by price tiers of brands**	**2006**	**2007–2008**	**2009**	**2011–2012**	**2013–2015**	**Change: 2007–2008 to 2013–2015**	***p*-value**	**Difference in change over 2007–2008 to 2013–2015 in comparison to Tier I cigarette brands**	***p*-value**
I	16.5%	18.3%	16.6%	12.8%	10.7%	−7.6%	0.0000		
II	13.1%	13.7%	13.4%	11.4%	8.9%	−4.8%	0.0000	2.8%	0.0350
III	10.0%	11.7%	11.0%	7.8%	6.4%	−5.2%	0.0000	2.3%	0.0290
IV	7.0%	9.7%	8.2%	5.1%	5.1%	−4.6%	0.0000	3.0%	0.0140
V	6.6%	7.2%	6.4%	4.7%	4.5%	−2.7%	0.0150	4.9%	0.0010

**Table 6:** Generalized Estimating Equations (GEE) estimation of trend in annual cigarette expenditure per smoker in China, 2006–2015.

	Coef.	Std. Err.	z	P>|z|	(95% Conf. Int.)	
2006	−898.48	67.45	−13.32	0.00	−1030.69	−766.28
2009	107.65	59.95	1.80	0.07	−9.84	225.15
2011–2012	133.24	62.72	2.12	0.03	10.31	256.17
2013–2015	20.79	73.61	0.28	0.78	−123.49	165.07
**Constant**	5133.12	138.86	36.97	0.00	4860.95	5405.29

Note: Coef. = Coefficient estimates; Std. Err. = Standard Error of Coefficient estimates; z = z statistic; P>|z| = *p*-value of z statistic; 95% Conf. Int. = 95% Confidence Interval of the Coefficient estimates. The reference survey wave is Wave 2 (2007–2008). Estimates were adjusted for age, gender, household income status, individual education and employment status, price tiers of cigarettes in last purchase, and city of residence of respondents.

**Table 7:** Generalized Estimating Equations (GEE) estimation of trend in cigarette expenditure share in smokers’ household income in China, 2006–2015.

	Coef.	Std. Err.	z	P>|z|	(95% Conf. Int.)	
2006	−0.01	0.00	−2.16	0.03	−0.02	0.00
2009	−0.01	0.00	−4.09	0.00	−0.02	−0.01
2011–2012	−0.03	0.00	−6.95	0.00	−0.03	−0.02
2013–2015	−0.05	0.00	−11.15	0.00	−0.06	−0.04
**Constant**	0.17	0.01	22.31	0.00	0.16	0.19

Note: Coef. = Coefficient estimates; Std. Err. = Standard Error of Coefficient estimates; z = z statistic; P>|z| = *p*-value of z statistic; 95% Conf. Int. = 95% Confidence Interval of the Coefficient estimates. The reference survey wave is Wave 2 (2007–2008). Estimates were adjusted for age, gender, household income status, individual education and employment status, price tiers of cigarettes in last purchase, and city of residence of respondents.

**Table 8:** Trend in annual cigarette expenditure per smoker in yuan in 2015 constant prices (predicted from GEE estimation) by age, gender, household income status, education, employment status, and price tier of brands in last purchase of smokers in China, 2007–2008 to 2013–2015.

**Cigarette expenditure by age group**	**2006**	**2007–2008**	**2009**	**2011–2012**	**2013–2015**	**Change: 2007–2008 to 2013–2015**	***p*-value**	**Difference in change over 2007–2008 to 2013–2015 in comparison to age group 18–35**	***p*-value**
18–35	1575	2618	2761	2736	2838	221	0.3010		
36–55	2065	2965	3157	3177	3003	38	0.6930	−183	0.4260
55+	1819	2673	2598	2664	2581	−92	0.4530	−313	0.1980
**Cigarette expenditure by gender**	**2006**	**2007–2008**	**2009**	**2011–2012**	**2013–2015**	**Change: 2007–2008 to 2013–2015**	***p*-value**	**Difference in change over 2007–2008 to 2013–2015 between male and female**	***p*-value**
Male	1958	2848	2972	2986	2883	35	0.6440		
Female	1109	2209	1960	2255	1948	−261	0.4110	−296	0.3620
**Cigarette expenditure by household income status**	**2006**	**2007–2008**	**2009**	**2011–2012**	**2013–2015**	**Change: 2007–2008 to 2013–2015**	***p*-value**	**Difference in change over 2007–2008 to 2013–2015 between low- and high-income**	***p*-value**
Low-income	1875	2774	2874	2903	2801	26	0.7380		
High-income	2568	3412	3662	3636	3465	54	0.8340	28	0.9170
**Cigarette expenditure by educational status**	**2006**	**2007–2008**	**2009**	**2011–2012**	**2013–2015**	**Change: 2007–2008 to 2013–2015**	***p*-value**	**Difference in change over 2007–2008 to 2013–2015 between low and high education**	***p*-value**
Low education	2051	2896	2941	3067	2899	3	0.9760		
High education	1836	2775	2929	2888	2811	36	0.7010	33	0.8180
**Cigarette expenditure by employment status**	**2006**	**2007–2008**	**2009**	**2011–2012**	**2013–2015**	**Change: 2007–2008 to 2013–2015**	***p*-value**	**Difference in change over 2007–2008 to 2013–2015 between not employed and employed**	***p*-value**
Not employed	1973	2772	2746	2800	2677	−95	0.3880		
Employed	1923	2878	3070	3085	2990	112	0.2370	207	0.1430
**Cigarette expenditure by price tiers of brands**	**2006**	**2007–2008**	**2009**	**2011–2012**	**2013–2015**	**Change: 2007–2008 to 2013–2015**	***p*-value**	**Difference in change over 2007–2008 to 2013–2015 in comparison to Tier I cigarette brands**	***p*-value**
I	4681	6475	6474	6234	5868	−607	0.0170		
II	2598	3376	4061	4087	3989	613	0.0060	1220	0.0000
III	1916	2668	2808	2827	2686	17	0.8710	625	0.0230
IV	1428	1975	1929	1897	2118	143	0.4210	750	0.0150
V	1825	1543	1714	1863	1825	282	0.3220	890	0.0200

**Table 9:** Trend in cigarette expenditure share in smokers’ household income (predicted from GEE estimation) by age, gender, household income status, education, employment status and price tier of brands in last purchase of smokers in China, 2007–2008 to 2013–2015.

**Cigarette expenditure share by age group**	**2006**	**2007–2008**	**2009**	**2011–2012**	**2013–2015**	**Change: 2007–2008 to 2013–2015**	***p*-value**	**Difference in change over 2007–2008 to 2013–2015 in comparison to age group 18–35**	***p*-value**
18–35	0.09	0.10	0.10	0.08	0.08	−0.02	0.0800		
36–55	0.13	0.13	0.11	0.10	0.07	−0.06	0.0000	−0.04	0.0030
55+	0.06	0.07	0.06	0.06	0.04	−0.03	0.0000	−0.01	0.4270
**Cigarette expenditure share by gender**	**2006**	**2007–2008**	**2009**	**2011–2012**	**2013–2015**	**Change: 2007–2008 to 2013–2015**	***p*-value**	**Difference in change over 2007–2008 to 2013–2015 between male and female**	***p*-value**
Male	0.11	0.11	0.10	0.09	0.06	−0.05	0.0000		
Female	0.05	0.09	0.05	0.07	0.05	−0.04	0.0190	0.00	0.7970
**Cigarette expenditure share by household income status**	**2006**	**2007–2008**	**2009**	**2011–2012**	**2013–2015**	**Change: 2007–2008 to 2013–2015**	***p*-value**	**Difference in change over 2007–2008 to 2013–2015 between low- and high-income**	***p*-value**
Low-income	0.11	0.12	0.10	0.09	0.07	−0.05	0.0000		
High-income	0.05	0.05	0.05	0.02	0.02	−0.03	0.0570	0.02	0.1450
**Cigarette expenditure share by educational status**	**2006**	**2007–2008**	**2009**	**2011–2012**	**2013–2015**	**Change: 2007–2008 to 2013–2015**	***p*-value**	**Difference in change over 2007–2008 to 2013–2015 between low and high education**	***p*-value**
Low education	0.12	0.13	0.11	0.11	0.07	−0.07	0.0000		
High education	0.09	0.09	0.09	0.07	0.06	−0.03	0.0000	0.03	0.0000
**Cigarette expenditure share by employment status**	**2006**	**2007–2008**	**2009**	**2011–2012**	**2013–2015**	**Change: 2007–2008 to 2013–2015**	***p*-value**	**Difference in change over 2007–2008 to 2013–2015 between not employed and employed**	***p*-value**
Not employed	0.14	0.14	0.12	0.11	0.08	−0.06	0.0000		
Employed	0.08	0.09	0.08	0.07	0.06	−0.04	0.0000	0.02	0.0030
**Cigarette expenditure share by price tiers of brands**	**2006**	**2007–2008**	**2009**	**2011–2012**	**2013–2015**	**Change: 2007–2008 to 2013–2015**	***p*-value**	**Difference in change over 2007–2008 to 2013–2015 in comparison to Tier I cigarette brands**	***p*-value**
I	0.16	0.18	0.15	0.13	0.11	−0.07	0.0010		
II	0.12	0.13	0.14	0.12	0.08	−0.04	0.0010	0.03	0.1600
III	0.10	0.11	0.10	0.08	0.06	−0.04	0.0000	0.03	0.0820
IV	0.09	0.10	0.08	0.07	0.06	−0.04	0.0000	0.03	0.0680
V	0.10	0.07	0.07	0.06	0.05	−0.02	0.1390	0.05	0.0280

## Appendix A

**Table A1:** Per capita annual household income by monthly household income categories reported in ITC China Surveys.

Reported Monthly Household Income Categories	2006	2007	2008	2009	2010	2011	2012	2013	2014	2015
	Mean Annual Household Income									
<1000	5067	5067	5067	5067		5067				
1000–2999	23,842	23,842	23,842	23,842		23,842				
3000–4999	46,540	46,540	46,540	46,540		46,540				
5000–6999	70,900	70,900	70,900	70,900		70,900				
7000–8999	94,566	94,566	94,566	94,566		94,566				
≥9000	111,008	124,729	146,664	168,363		208,160				
	**Mean Per Capita Annual Household Income**									
<1000	1960	1880	1817	1909		1892	1892	1892	1892	1892
1000–2999	8117	8025	7510	7932		7896	7896	7896	7896	7896
3000–4999	14,919	14,923	13,965	14,752		14,640	14,640	14,640	14,640	14,640
5000–6999	22,571	22,110	20,788	21,676		21,786	21,786	21,786	21,786	21,786
7000–8999	28,368	28,204	26,790	26,922		27,571	27,571	27,571	27,571	27,571
≥9000	32,139	37,842	34,677	47,199		64,861	72,902	81,698	90,822	99,743
Per capita household income growth (%)	10.7%	12.2%	8.4%	11.5%	8.1%	8.3%	9.5%	9.2%	9.0%	8.2%
Inflation (%)	1.5%	4.8%	5.9%	-0.7%	3.3%	5.4%	2.6%	2.6%	2.0%	1.5%

Source: Authors’ calculations based on data from China Household Finance Survey, 2011 [35]; IMF World Economic Outlook Database [36]; National Bureau of Statistics of China [25]; and ITC China Survey Waves 1–5. Notes: The mean per capita annual household income values for the lower five income groups for the years 2012–2015 were not adjusted for inflation or income growth. For the open-ended top income group with monthly household income 9000 yuan (1445 USD) and above, the mean per capita household income (64,861 yuan or 10,038 USD) imported from the CHFS 2011 was adjusted for inflation and per capita income growth for the years 2012–2015 to obtain the nominal income values for respective years. 1 USD was equivalent to 9.97 yuan in 2006, 7.61 yuan in 2007, 6.95 yuan in 2008, 6.83 yuan in 2009, 6.77 yuan in 2010, 6.46 yuan in 2011, 6.31 yuan in 2012, 6.20 yuan in 2013, 6.14 yuan in 2014 and 6.23 yuan in 2015 [37].

**Table A2:** Per capita income classification of urban households in China, 2006–2015.

	Per Capita Total Income of Urban Households (yuan)	Per Capita Total Income of Urban Households, Low Income Households (second decile group) (yuan)	Per Capita Total Income of Urban Households, Lower Middle-income Households (second quintile group) (yuan)	Per Capita Total Income of Urban Households, Middle-income Households (third quintile group) (yuan)	Per Capita Total Income of Urban Households, Upper Middle-income Households (fourth quintile group) (yuan)	Per Capita Total Income of Urban Households, High-income Households (ninth decile group) (yuan)	Per Capita Monthly Total Income Threshold of Urban High-income Households (yuan)	Per Capita Monthly GDP (yuan)
	(1)	(2)	(3)	(4)	(5)	(6)	(7)	(8)
2006	12,719	5946	8104	11,052	15,200	20,700	1725	1395
2007	14,909	6993	9568	12,979	17,685	24,107	2009	1709
2008	17,068	7917	10,975	15,055	20,784	28,519	2377	2010
2009	18,858	8957	12,345	16,858	23,051	31,172	2598	2185
2010	21,033	10,247	13,971	18,921	25,498	34,255	2855	2573
2011	23,979	11,751	15,881	21,440	29,059	39,216	3268	3034
2012	26,959	13,725	18,375	24,531	32,759	43,471	3623	3334
2013	-	-	-	-	-	-	3971	3654
2014	-	-	-	-	-	-	4274	3934
2015	-	-	-	-	-	-	4550	4188

Source: Per capita total income of urban households from National Bureau of Statistics (NBS) China [25]; per capita Gross Domestic Product (GDP) from World Development Indicators Database, The World Bank [37]. Notes: The per capita income and GDP are not adjusted for inflation. Per capita monthly total income threshold of urban high-income households for 2006–2012 in column (7) was calculated by dividing per capita total income of urban households in the ninth decile group in column (6) by 12. Prior to 2013, the NBS conducted the urban and rural household surveys separately. Since 2013, the NBS started an integrated urban and rural households survey on income and expenditure and living conditions. The coverage, methodology, and definitions used in the surveys after 2013 are different from those used for separate urban and rural household surveys until 2012. The income classification available from the NBS is therefore not comparable for periods before and since 2013. So, for 2013–2015, we calculated the income thresholds in column (7) by adjusting the per capita monthly total income threshold of urban high-income households from 2012 using the same growth rate as per capita GDP. 1 USD was equivalent to 9.97 yuan in 2006, 7.61 yuan in 2007, 6.95 yuan in 2008, 6.83 yuan in 2009, 6.77 yuan in 2010, 6.46 yuan in 2011, 6.31 yuan in 2012, 6.20 yuan in 2013, 6.14 yuan in 2014, and 6.23 yuan in 2015 [37].
